# Enhancing Conductivity and Self-Healing Properties of PVA/GEL/OSA Composite Hydrogels by GO/SWNTs for Electronic Skin

**DOI:** 10.3390/gels9020155

**Published:** 2023-02-15

**Authors:** Xiaohu Chen, Haonan Zhang, Jiashu Cui, Yanen Wang, Mingyang Li, Juan Zhang, Changgeng Wang, Zhisheng Liu, Qinghua Wei

**Affiliations:** 1 Department of Indurstry and Engineering, School of Mechanical Engineering, Northwestern Polytechnical University, Xi’an 710072, China; 2 Bio-Additive Manufacturing University-Enterprise Joint Research Center of Shaanxi Province, Northwestern Polytechnical University, Xi’an 710072, China

**Keywords:** conductivity, self-healing properties, mechanical properties, biocompatibility, hydrogel electronic skin

## Abstract

The use of flexible, self-healing conductive hydrogels as a type of typical electronic skin with the function of transmitting sensory signals has attracted wide attention in the field of biomaterials. In this study, composite hydrogels based on polyvinyl alcohol (PVA), gelatin (GEL), oxidized sodium alginate (OSA), graphene oxide (GO), and single-walled carbon nanotubes (SWNTs) were successfully prepared. The hydrogen and imine bonding of the composite hydrogels gives them excellent self-healing properties. Their self-healing properties restore 68% of their breaking strength and over 95% of their electrical conductivity. The addition of GO and SWNTs enables the PGO-GS hydrogels to achieve a compressive modulus and conductivity of 42.2 kPa and 29.6 mS/m, which is 8.2 times and 1.5 times that of pure PGO, respectively. Furthermore, the PGO-GS hydrogels can produce profound feedback signals in response to deformation caused by external forces and human movements such as finger flexion and speech. In addition, the PGO-GS hydrogels exhibit superior biocompatibility compared to PGO. All of these results indicate that the PGO-GS hydrogels have great potential with respect to future applications in the field of electronic skin.

## 1. Introduction

The skin is the largest organ of the human body, and has the characteristics of flexibility, self-repairability, and tactile sensitivity [1,2,3]. Bionic tactile sensor devices that mimic the characteristics and functions of human skin are referred to as electronic skin [4]. With the rapid development of electronic technology in recent years, increasingly more electronic devices are developing towards the directions of achieving miniaturization, flexibility, and lightweight properties [5,6]. Electronic skin has also been widely used in wearable devices, health monitoring, intelligent robots, and bionic prostheses [7,8,9,10].

In order to perceive deformations in real time, electronic skin must be highly flexible and resilient, and should be able to generate specific response signals to stimuli and provide timely feedback [11,12]. Conductive hydrogels are soft materials with good flexibility and biocompatibility, whose structure is similar to natural living tissue [13,14,15]. Conductive hydrogels are considered to constitute an ideal material for the preparation of bionic electronic skin due to their functional designability [16,17,18]. Bionic electronic skin requires a high level of electrical conductivity and sensitivity, which are often achieved by introducing conductive filling materials [19]. Examples of such incorporations include the addition of conductive polymers (polypyrrole (PPy) and polyaniline (PANI)) and inorganic nanomaterials (carbon nanotubes, graphene, and metal nanoparticles) [13,20,21,22]. Among them, graphene and carbon nanotubes have excellent electrical, mechanical, and surface properties, and have shown the greatest potential for use in bionic electronic skin [23,24]. Compared with conductive polymers, the excellent mechanical properties of graphene and carbon nanotubes can improve the overall mechanical properties (especially the toughness) of hydrogels, while building stable conductive networks within the hydrogels [25,26,27]. For example, Wu et al. [28] prepared polyvinyl alcohol (PVA)/graphene oxide (GO) composite hydrogels by introducing metal ion coordination bonds. The tensile strength of the PVA-GO hydrogel could reach 11.10 MPa, which is 175% higher than that of pure PVA. Wu et al. [29] successfully prepared a composite hydrogel based on PVA, carboxymethyl chitosan (CMCS), oxidized sodium alginate (OSA), and oxidized multi-walled carbon nanotubes (OMWCNTs). The addition of OMWCNTs increased the fracture strength and electrical conductivity of the hydrogel to 0.8 MPa and 70.2 mS/m, respectively, which were 2.5 and 4 times higher than those of the previous hydrogel. The change in the resistance of the composite hydrogel after 200 cycles of 20% constant strain was almost identical to the initial value, thus demonstrating the excellent stability of the composite hydrogel. Li et al. [30] successfully constructed gelatin (GEL)-OSA-PVA ternary hydrogels by mixing PVA with GEL and OSA. By adjusting the concentration of PVA and the degree of oxidation of OSA, the hydrogel obtained adjustable mechanical properties with a maximum compressive modulus of 75 kPa. In the 300 s fatigue compression test, a large amount of PVA only reduced the stress of the hydrogel by 30.99%. In addition, the hydrogel exhibits good electrical conductivity (10.68 mS/m) due to the presence of free ions.

In addition to the mechanical and electrical properties mentioned above, the self-healing properties and biocompatibility of hydrogels are also of interest. Self-healing hydrogels can spontaneously repair damage without any external stimulation, which can effectively extend the lifetime of hydrogel electronic skins and expand their applications in extreme environments [31,32]. Self-healing is mainly achieved by constructing reversible dynamic bonds (non-covalent and dynamic covalent bonds) in hydrogel networks. The non-covalent bonds include hydrogen bonds, metal coordination bonds, etc. Dynamic covalent bonds include imine/hydrazone bonds, Diels–Alder reactions, borate ester bonds, and disulfide bonds [33,34]. In addition, electronic skin needs to be biocompatible to avoid damaging human skin when employed as health-monitoring devices [25,35]. Therefore, a kind of hydrogel with excellent properties (conductivity, mechanics, self-healing, biocompatibility, etc.) used for electronic skin still needs to be found, and this pursuit is attracting a considerable amount of attention.

Herein, we report the construction of a conductive and self-healing composite hydrogel using PVA, GEL, OSA, GO, and single-walled carbon nanotubes (SWNTs). The formation of imine bonds between GEL and OSA allows the hydrogel to heal itself without any external stimulation. Two kinds of nanomaterials, GO and SWNTs, were applied to enhance the properties of the composite hydrogel, such as effecting a higher compression modulus, better elastic behavior, and enhanced electrical conductivity. Finally, the prepared composite hydrogel proved to have excellent biocompatibility and sensitivity. These characteristics show that the prepared composite hydrogel has great potential in application scenarios regarding electronic skin such as wearable devices, health monitoring, and voice recognition.

## 2. Results and Discussion

### 2.1. Microtopography of Hydrogels

**Figure 1:**
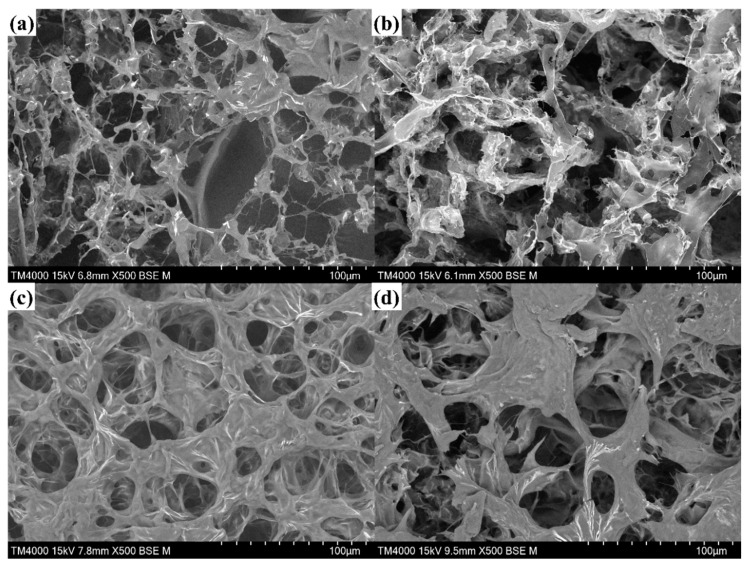
The SEM images of (**a**) PGO hydrogel, (**b**) PGO-GS1 hydrogel, (**c**) PGO-GS3 hydrogel, and (**d**) PGO-GS5 hydrogel.

As shown in Figure 1, which presents scanning electron microscopy (SEM) images of the freeze-dried PGO and PGO-GA hydrogels, it can be seen that all the freeze-dried hydrogels show the characteristics of a porous structure. Particularly, the GO sheets and SWNTs were evenly distributed without agglomeration in the PGO-GS3 hydrogel framework (Figure 1c). Compared with the PGO hydrogel, the PGO-GS3 hydrogel has a porous three-dimensional network structure and more uniform pore size, which is conducive to the conductivity and mechanical strength of hydrogels.

The density and porosity of the hydrogels were also calculated, as shown in Table 1. Notably, the PGO-GS3 hydrogel has the highest porosity and the lowest density; this is because they are closely related to the structure of the hydrogel. This result is consistent with the results observed using electron microscopy.

Figure S1 shows the swelling ratios of the hydrogels. The swelling ratio of the PGO hydrogel reaches 13.91, which is significantly higher than that of the PGO-GS hydrogel. This indicates that the PGO hydrogel has large internal pores and loose structures, and this finding is consistent with the SEM images. The reason behind this is that the addition of GO and SWNTs may increase the viscosity of the hydrogel, resulting in a denser network, and the denser hydrogel network prevents water molecules from diffusing into the hydrogel.

### 2.2. Chemical Structure of the Hydrogels

**Figure 2:**
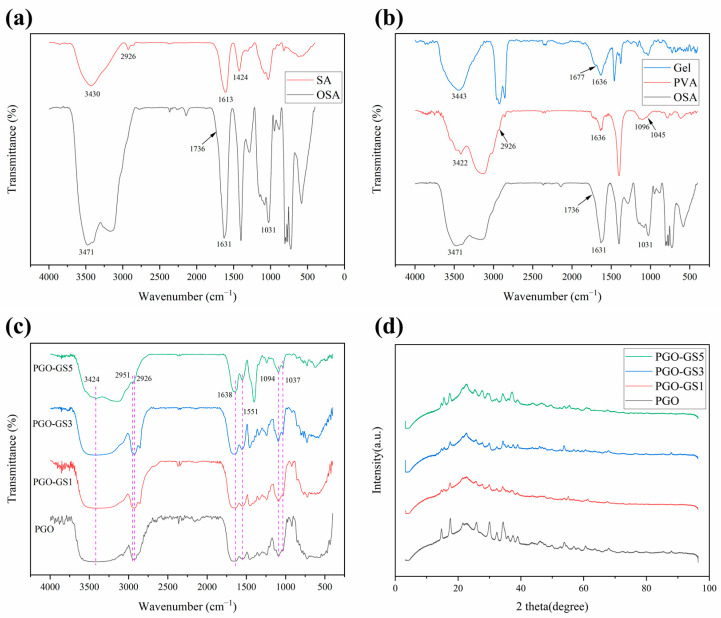
The FTIR spectrum of (**a**) SA and OSA; (**b**) GEL, PVA, and OSA; (**c**) PGO and PGO-GS hydrogels. (**d**) The XRD patterns of PGO and PGO-GS hydrogels.

The spectra of SA and OSA are shown in Figure 2a. The peak located at 1736 cm^−1^ in OSA spectra is the characteristic peak of -CHO [29,36], which is caused by the symmetric stretching vibration of C=O, indicating that the oxidation reaction was successful. Meanwhile, the peak of SA at 3430 cm^−1^ is wider than that in OSA, which also indicates that the content of -OH decreased in OSA.

Figure 2b shows the spectra of PVA and GEL. The peak at 3422 cm^−1^ in the PVA spectrum was generated by hydrogen bonding between hydroxyl groups. The C-O stretching vibration in the crystalline region and amorphous region of PVA produced peaks at 1096 cm^−1^ and 1045 cm^−1^, respectively. In GEL’s spectrum, the stretching vibration and deformation vibration of -NH_2_ are at the 3443 cm^−1^ and 1677 cm^−1^ peaks, respectively.

Figure 2c shows the spectrum of the PGO hydrogel with different GO and SWNTs fractions. The enhancement peak at 3424 cm^−1^ was caused by the overlap of hydrogen bonds. The peak of PGO-GS5 at 3424 cm^−1^ is significantly weaker than the other samples. This may be attributable to the high content of GO and SWNTs, which leads to a certain degree of agglomeration of the two materials, thus affecting the formation of hydrogen bonds in the hydrogel. The peaks at 1636 cm^−1^ and 1547 cm^−1^ belong to C=N vibration, which occurred due to the formation of imine bonds. The peak at 1094 cm cm^−1^ is stronger than that at 1037 cm^−1^, indicating that PVA has higher crystallinity and forms hydrogen bonds with the other two macromolecules. The peak at 1094 cm^−1^ of the PGO-GS5 spectrum is weak, which further indicates that high GO and SWNTs fractions will hinder the formation of hydrogen bonds between three macromolecules. In general, the addition of GO and SWNT does not significantly change the composition of the organic functional groups in the PGO hydrogel.

The formation of the PGO-GS hydrogel was also analyzed by XRD. Figure 2d shows the XRD patterns of the hydrogels with different GO and SWNTs fractions. PGO-GS formed a low-intensity diffraction peak at 2θ = 15.4°, which is the characteristic peak of GO. The curve shows distinct peaks at 2θ = 15°, 22°, and 34°, which are typical XRD patterns for OSA [29]. The XRD pattern of the PGO and PGO-GS hydrogel shows a wide, amorphous diffraction peak centered at 2θ = 22.7°. It is well known that GEL and PVA express their ordered structure at around 2θ = 20° [28,37,38], and that amorphous PVA phase produces a weak band at 2θ = 42° [39]. The PGO-GS composite hydrogel has a weaker diffraction peak at 2θ = 14.75° than the PGO hydrogel, indicating that the addition of GO and SWNTs attenuates the crystallization of PVA. In addition, there is no significant difference between the PGO and PGO-GS hydrogels. All the samples have similar curves, which shows that the incorporation of GO sheets and SWNTs has no obvious effect on the crystallinity of PVA and GEL. It has been confirmed that there is no significant phase separation appearing in this system, indicating that GO and SWNTs are well dispersed in the PGO-GS hydrogel.

### 2.3. Mechanical Properties of Hydrogels

**Figure 3:**
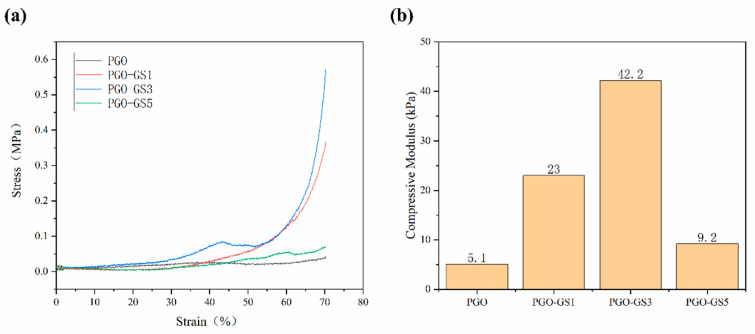
(**a**) The compression stress–strain curves of PGO and PGO-GS hydrogels. (**b**) The compressive modulus of PGO and PGO-GS hydrogels.

The mechanical strength of the hydrogels was further quantified with compressive tests. The compressive stress–strain curves of the PGO and PGO-GS hydrogels are shown in Figure 3a. It can be seen that the mechanical properties of the PGO-GS hydrogel are better than those of the PGO hydrogel. This is because the sheet-like shape of the GO molecule, tubular shape of the SWNT molecule, and three kinds of macromolecular chains are intertwined to form a tighter, more stable network structure thanks to hydrogen bonding. During the compression process, part of the compression stress was transferred to the GO and SWNTs dispersed in the hydrogel, ultimately increasing in the compression strength of the PGO-GS hydrogel. However, the compressive strength of PGO-GS5 shows a decrease compared to PGO-GS3 (Figure 3b). This is mainly due to the agglomeration of GO and SWNT caused by the electrostatic force and van der Waals forces between the nanoparticles, resulting in a decrease in the mechanical properties of the hydrogel [40,41]. Notably, the addition of GO and SWNTs resulted in a compressive modulus of 42.2 kPa for the PGO-GS3 hydrogel, which is as much as eight times higher than that of PGO.

### 2.4. Conductive Properties of Hydrogels

**Figure 4:**
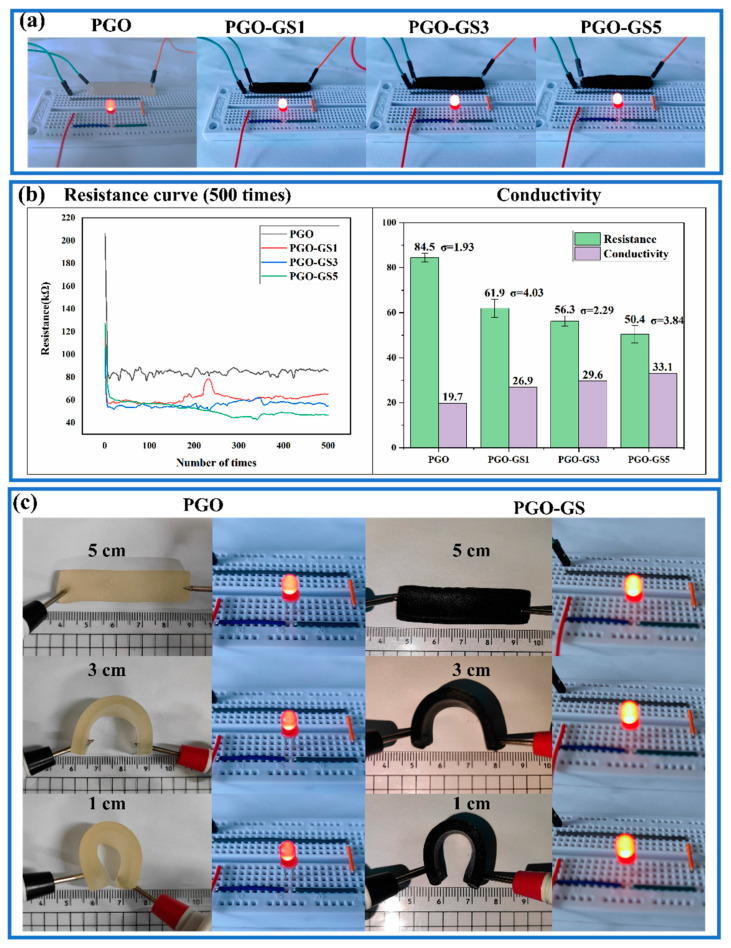
(**a**) The brightness of LED when hydrogel was connected to the circuit. (**b**) The conductivity and resistance of hydrogels. (**c**) The brightness of the LED when bending the hydrogel. (The applied voltage of all displayed circuits is 10 V.)

Figure 4a shows the brightness of an LED when the hydrogel was connected to the displayed circuit. It can be seen that the conductivity of the PGO hydrogel improves with the increasing content of GO and SWNTs. Figure 4b shows the resistance curves for 500 measurements of the different hydrogels. It can be seen that the resistance of the hydrogel measured when it was first connected to the circuit has a large deviation. Therefore, the resistance values of the first 100 measurements were discarded, and only the last 400 measurements were kept to calculate the mean value and determine the standard deviation in this paper (the subsequent resistance measurements are treated in the same way). The conductivity of the PGO-GS3 hydrogel reached 29.6 mS/m, which is 1.5 times that of the PGO hydrogel. This is mainly attributed to the conductive network formed by the GO sheets and SWNTs inside the hydrogel. The increasing content of GO and SWNTs can significantly promote the density of the conductive network, resulting in higher conductivity. However, when the sum of the two fillers divided by the mass of GEL is more than 4.5%, the growth rate of the conductivity is significantly reduced. This phenomenon may be caused by the uneven dispersion of GO and SWNTs.

Interestingly, we found that when the wires were placed on either side of the PGO and PGO-GS hydrogels, the brightness of the LED would gradually darken with the folding of the hydrogel (Figure 4c). Therefore, the electrical conductivity of the hydrogel was also tested at different degrees of bending (Table S1). When the distance between the two electrodes decreased from 5 cm to 1 cm, the conductivity of the PGO hydrogel decreased from 19.11 to 18.73 mS/m, and the conductivity of the PGO-GS3 hydrogel decreased from 29.13 to 26.60 mS/m. The variation range of the PGO-GS3 hydrogel is about 8.6%, which demonstrates the PGO-GS3 hydrogel’s potential as a flexible, electronic sensing skin.

### 2.5. Self-Healing Property of Hydrogels

It can be seen that the PGO-GS3 hydrogel has superior comprehensive properties by analyzing the swelling, mechanical, and conductive properties of the different hydrogels. Therefore, the PGO-GS3 hydrogel was selected to evaluate the self-healing performance and other subsequent performances and applications.

**Figure 5:**
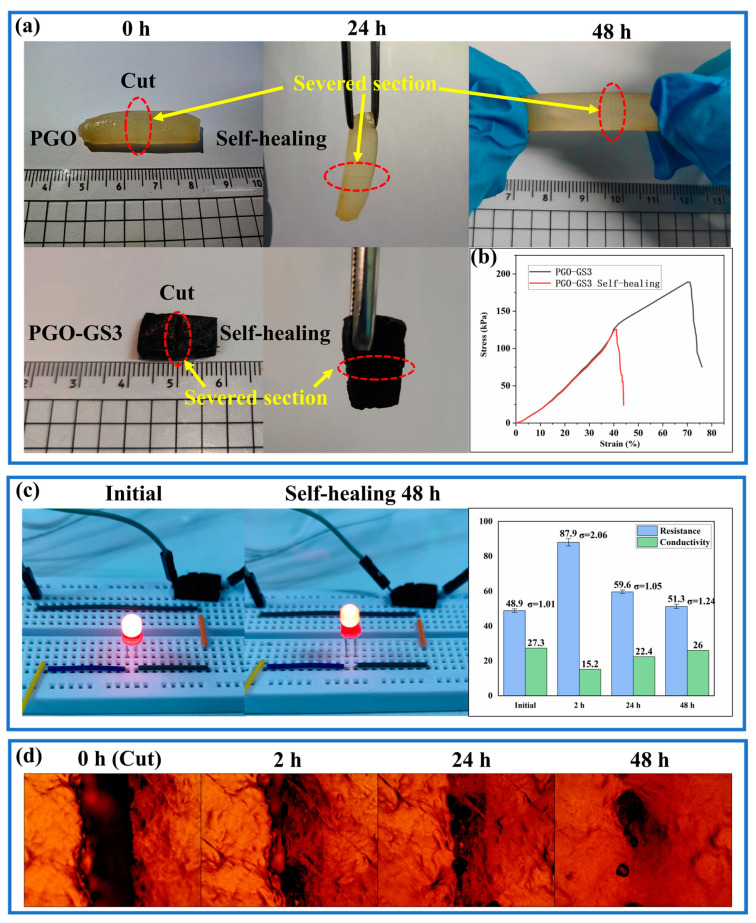
(**a**) Photos of the self-healing process of hydrogels. (**b**) The tensile curves of the initial hydrogel and the 48 h self-healing hydrogel (PGO-GS3). (**c**) Conductivity of initial hydrogel and the 48 h self-healing process of the hydrogel (PGO-GS3). (**d**) The self-healing process of the PGO hydrogel under optical microscope.

Figure 5a shows that both the PGO and PGO-GS3 hydrogels have good self-healing properties. After 24 h of self-healing, both hydrogels were able to bear their own weights. After 48 h, although the cut marks were still visible, the hydrogels had completely healed and could withstand various tensile forces.

The typical stress–strain data of the original PGO-GS3 hydrogel and the self-healing hydrogel are shown in Figure 5b. After 48 h of healing, the fracture strength and elongation at break reached 0.13 MPa and 40.9%, respectively, which were 68% and 57% of the initial hydrogel (0.19 MPa and 71.2%). This is the result of hydrogen bonds and imine bonds formed between the molecular chains of PVA and GEL on the fracture’s surface.

The conductivity-related self-healing performance of the hydrogels was also verified, as shown in Figure 5c. Although the brightness of the LED decreased slightly after 48 h of self-healing, its conductivity recovered to more than 95% of the initial value. This also suggests that the imine bonds and hydrogen bonds help the hydrogel reconstruct the network structure during the self-healing process.

For further investigation, the PGO hydrogel was selected to observe the self-healing process under an optical microscope (Figure 5d). It can be seen that most of the gaps between the cut areas of the PGO hydrogel disappeared after 48 h of self-healing.

### 2.6. Electronic Skin

**Figure 6:**
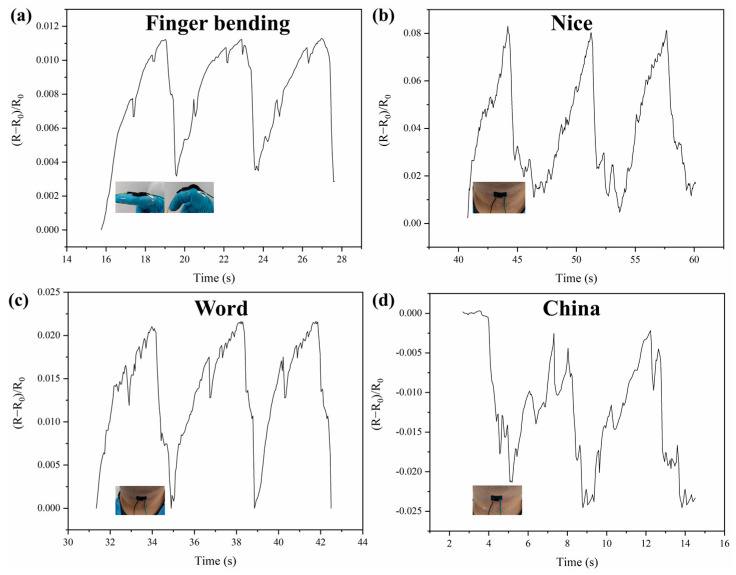
Sensing signals of encapsulated hydrogel corresponding to (**a**) finger bending, (**b**) the vocalization of the word “Nice”, (**c**) the vocalization of the word “LOVE,” and (**d**) the vocalization of the word “China”.

To explore the ability of the PGO-GS3 hydrogel to monitor human movement and recognize speech, the PGO-GS3 hydrogel was attached to the fingers and throat of a subject to detect the signals generated by finger bending and speaking. Figure 6a shows the real-time resistance change of the hydrogel during finger bending. Obviously, the bending of fingers can sufficiently stimulate the hydrogel so as to generate regular electrical signals. In addition, the throat vibrations caused by human speech can also be accurately captured (Figure 6b–d). It can be seen from the comparison that the feedback signal of the PGO-GS3 hydrogel is different for different words, which proves that the PGO-GS3 hydrogel has the potential to be used in speech recognition.

### 2.7. Cytocompatibility

A CCK8 assay was performed to determine the L929 cells’ viability after being cultured in 10% hydrogel leach liquor solution. The experimental results are shown in Table 2.

Although the survival rate of the cells cultured in 10% leach liquor solution is lower than that of the control group, the cell survival rates in both groups are much higher than 50% after 48 h of incubation. This indicates that both the PGO and PGO-GS3 hydrogels have good cell capability.

As a comparison, the cell survival rate of PGO-GS3 group (95.3%) was significantly higher than that of PGO experimental group (75.8%). This indicates that the addition of GO and SWNTs has a potential effect on cell proliferation. In this study, the activity levels may be related to the good mechanical properties and porosity of the PGO-GS3 hydrogel.

Overall, the above biological evaluations prove that the PGO-GS3 hydrogel possess good potential for use as a biomaterial for flexible bionic electronic skin that does not cause cellular inflammation.

## 3. Conclusions

In this study, a series of conductive composite hydrogels were successfully constructed by introducing GO and SWNTs into a PVA/GEL/OSA ternary hydrogel system. The SA was successfully oxidized and formed dynamic imine bonds with the GEL, which gave the hydrogel good self-healing properties. The obtained self-healing properties restored 68% of the hydrogels’ original breaking strength and over 95% of their electrical conductivity. The addition of PVA effectively improves the toughness of the hydrogels, and the introductions of GO and SWNTs effectively enhance the electrical and mechanical properties of the hydrogels. The compressive modulus and electrical conductivity of the PGO-GS hydrogel are 42.2 kPa and 29.6 mS/m, respectively, i.e., 8.2 and 1.5 times higher than those of pure PGO. In addition, the cytocompatibility tests not only demonstrated the good biocompatibility of the PGO-GS hydrogels but also showed that GO and SWNTs have a potentially positive effect on cell proliferation. Notably, PGO-GS conductive hydrogels can quickly capture external stimulations, including human movement and speech, and give correspondingly accurate and timely electrical signal feedback. Compared to similar studies [29,30,37], PGO-GS hydrogels have better self-healing properties (conductivity and tensile properties), higher electrical conductivity, and better biocompatibility. These properties indicate that PGO-GS hydrogels have promising applications in the field of electronic skin, such as voice recognition, human movement, and health monitoring.

## 4. Materials and Methods

### 4.1. Materials

GO and SWNTs were purchased from Jiacai Technology Co., Ltd. (Chengdu, China). Sodium alginate (SA) (1.05~1.15 Pa·S) was purchased from Fuchen Chemical Reagent Co., Ltd. (Tianjin, China). GEL (BR, jelly strength ≥ 220) was purchased from Qiansheng Biotechnology Co., Ltd. (Hefei, China). Sodium chloride (NaCl) (AR) was purchased from Sinopharm Chemical Reagent Co., Ltd. (Beijing, China). PVA (polymerization degree 1799, 98% alcoholysis), Ethanol (C_2_H_6_O) (99.7%), Sodium periodate (NaIO_4_, AR, 99.5%), and Ethylene glycol (C_2_H_6_O_2_) (GC, >99%) were obtained from McLean Biochemical Technology Co., Ltd. (Shanghai, China). Potassium bromide (KBr) was purchased from Tianguang Optical Instrument Co., Ltd. (Tianjin, China). Deionized (DI) water was obtained using the pure water machine in our laboratory.

### 4.2. Preparation of OSA

**Figure 7:**
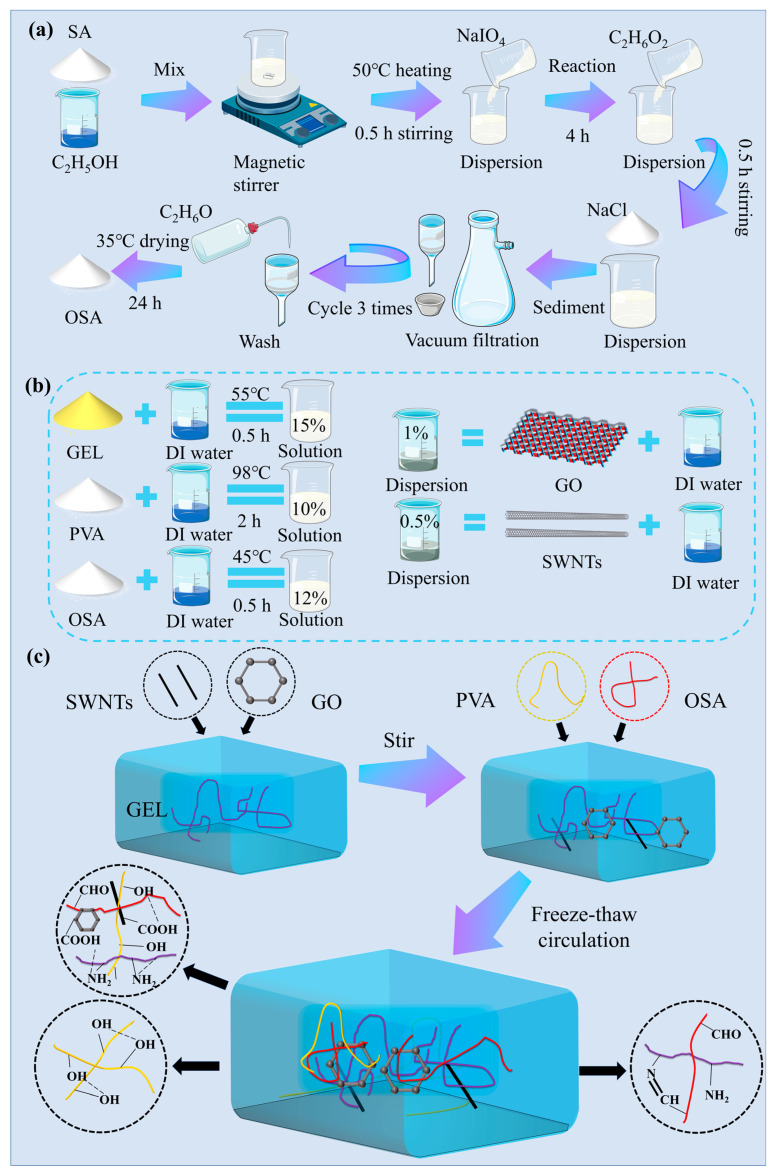
(**a**) Preparation process of OSA. (**b**) Mass fraction of each component solution of composite hydrogel. (**c**) Preparatory process for PGO-GS hydrogel.

The oxidation of SA was carried out according to a previously reported method, as shown in Figure 7a [42,43]. In brief, SA (10 g) was dispersed in ethanol (50 mL) in a water bath at 40 °C. Then, 50 mL NaIO_4_ solution (5 g) was added into the SA solution to react for 4 h, which was put on a magnetic stirrer in the dark at 25 °C. Then, 7.5 mL of C_2_H_6_O_2_ and 5 g of NaCl were added and quenched for 0.5 h under stirring. The sediment was filtered out through a vacuum filtration device. The final product was washed with C_2_H_6_O three times and then vacuum-dried at 35 °C for 24 h to obtain OSA.

### 4.3. Preparation of Hydrogel

In order to prepare PVA–GEL–OSA–GO–SWNT hydrogels with different compositions (Figure 7b,c), GEL (15 wt %) was dissolved in DI water at 55 °C and stirred for 0.5 h. The prepared OSA (12 wt %) was dissolved in DI water at 45 °C and stirred for 0.5 h. PVA (10 wt %) was dispersed in DI water in a 98 °C water bath with stirring for 2 h to form a solution. After defoaming at 20 °C for 5 h, 10 mL PVA solution was added to 10 mL GEL solution; then, the mixed solution was magnetically stirred at 40 °C for 0.5 h. Subsequently, the same volumes of GO (1 wt %) and SWNT (0.5 wt %) dispersions were added to PVA–GEL mixed solution in corresponding proportions and stirred for 1 h. Finally, 10 mL of OSA solution was added to the above mixed solution and stirred at 40 °C for 0.5 h. The final solution was poured into a mold and left at 20 °C for 1.5 h. Then, the mold was frozen at −20 °C for 12 h and thawed at 20 °C for 2 h, which is known as a freeze–thaw cycle. The freeze–thaw cycle was repeated 3 times to obtain PVA–GEL–OSA–GO–SWNT hydrogel. The hydrogel is named PGO-GSx, where x represents the mass fraction of GO relative to gelatin. Pure PVA–GEL–OSA hydrogel was prepared by the same method (it was named PGO hydrogel).

### 4.4. Characterization

#### 4.4.1. Constituent Analysis

FT-IR spectroscopic measurements were performed using an infrared spectrometer (ALPHA II, BRUKER, Salbruken, GER). The hydrogel sample was placed in an oven (DGG-9140A; LINPIN, Shanghai, CHN) at 35 °C for 36 h to dehydrate it completely. Then, the sample was placed into an agate mortar and ground into powder by adding liquid nitrogen. Finally, the FT-IR spectrum of the sample was analyzed using the KBr method [44].

The diffraction patterns of PGO-GS hydrogels were detected by an X-ray diffractometer (D8 DISCOVER A25, BRUKER, Salbruken, GER) using Co K radiation. The range of 2θ was 5~90°. The scanning rate was 0.1°/s, and the scanning step was 0.02°.

#### 4.4.2. Structural Analysis

The surface morphologies of hydrogels were observed by a scanning electron microscope (SEM) (TM4000PLUS, HITACHI, Tokyo, Japan). The hydrogel samples were freeze-dried for 24 h with a freeze dryer (TF-FD-27, Tianfeng, Shanghai, China), and then the surfaces of the samples were sprayed with gold for observation.

The density of hydrogels was calculated by dividing the mass of the freeze-dried hydrogel by its volume. Cylindrical hydrogels were obtained by molding and freeze-drying for 24 h. The diameter and height of each hydrogel were measured, using a vernier caliper to calculate the volume. Each sample was measured 5 times and the mean value was reported.

The porosity of hydrogels was measured according to the alcohol displacement method [45]. Briefly, the freeze-dried hydrogels were weighed (*W_d_*) and immersed in anhydrous ethanol at 25 °C for 12 h. The wet hydrogels (*W_w_*) were then weighed again. The porosity was determined by the following equation:

$$\mathrm{Porosity}=\frac{W_{w}-W_{d}}{\rho V}$$

where *ρ* is the absolute ethyl alcohol density (0.789 g/cm^3^), and *V* is the volume of freeze-dried hydrogel.

The swelling behavior of PGO-GS hydrogel was studied in phosphate buffer solution (PBS) at 25 °C. The freeze-dried hydrogels were immersed in the PBS (pH = 7.4) for 24 h. After the sample was taken out, the water on the swollen hydrogel’s surface was wiped off with absorbent paper, and then the hydrogel was weighed with an electronic balance (PKM124ZH; OHAUS, Pine Brook, NJ, USA). The swelling ratio was calculated by the following equation:

$$\mathrm{Swelling}\;\mathrm{ratio}=\left(W_{s}-W_{d}\right)/W_{d}$$

where *W_d_* and *W_s_* are the weights of dried and swollen samples, respectively.

#### 4.4.3. Mechanical Performance

The mechanical tests were carried out using a universal testing machine (CTM2500; Liangong, Dezhou, Shandong Province, China). The compressive mechanical properties of PGO-GS hydrogels were measured with cylindrical samples (H = 50 mm and d = 10 mm). The compressive speed was set as 1 mm/min and the compressive modulus was calculated from the approximate linear fitting values of the stress–strain curves within the strain range of 30~40%. The tensile test used to evaluate the self-healing property of PGO-GS3 was carried out with a rectangular sample (L = 50 mm, W = 12 mm, and H = 5 mm) at a speed of 5 mm/min.

In the previous specifications, L is the length of the hydrogel, W is the width of the hydrogel, H is the height of the hydrogel, and d is the diameter of the hydrogel. The meanings of the three letters provided below are the same.

#### 4.4.4. Conductive Property Measurement

The conductivity of the hydrogel was determined through observing the brightness of an LED bulb in the entire circuit when the hydrogels with different deformations were connected. The circuit was powered by DC stabilized constant current power supply (SPD3303C, Dingyang Technology, Shenzhen, Guangdong Province, CHN).

The electric resistance of the hydrogel (L = 50 mm, W = 8 mm, and H = 4 mm) was tested with a digital source meter (Keithley 6500, Beaverton, OR, USA). The conductivity (*σ*) of the sample was calculated using the following formula:

$$\sigma =\frac{l}{Rs}$$

where *l* and *s* are the length and cross-sectional area of the hydrogel sample, respectively. *R* is the measured resistance.

#### 4.4.5. Self-Healing Behaviors

In order to study the self-healing behavior of PGO-GS hydrogels at room temperature, the hydrogel samples were cut into two halves using a knife. Then, the two hydrogels were placed together without any external stimulation. After 48 h, the tensile stress–strain curves of the self-healing PGO-GS3 hydrogels (L = 20 mm, W = 12 mm, and H = 5 mm) were measured again with a universal testing machine. The self-healing process of the PGO hydrogel at different time points was observed using an orthostatic metallographic microscope (9XB-PC, Shangguang, Shanghai, CHN). The PGO-GS3 hydrogel is black and thus could not be observed under an optical microscope.

#### 4.4.6. Cytocompatibility Test

Mouse epithelioid fibroblast cells (L929) were used to evaluate the biocompatibility of the hydrogels. All cell culture-related reagents were purchased from Bojin Biotechnology Co., Ltd. (Xi’an, Shaanxi Province, China).

Both PGO and PGO-GS3 hydrogel pieces (L = 5 mm, W = 5 mm, and H = 5 mm) were placed in a glass petri dish and sterilized under ultraviolet radiation for 4 h. Then, one part of PGO and PGO-GS3 hydrogels was immersed in 10 mL PBS for 48 h to extract the leach solution.

To test CCK8, 4 testing groups were created and each group included 10 wells of a 96-well plate. No cells were seeded in the first group for blank well absorption value measurements. The cells were seeded at a density of 7 × 10^4^ cells/mL per well in the second, third, and fourth groups. For all groups, 90 μL cell media (first group) or cell suspension (other groups, ~6 × 10^3^ cells per well) were added in each well. For each well in all groups, each surrounding well was filled with 100 μL of PBS if it was empty. This ensured that all the wells in all groups were surrounded by wells filled with the same amount of solution. The cells were incubated at 5% CO_2_ and 37 °C for 12 h for cell attachment. Then, the 10 μL PBS leach liquors of PGO and PGO-GS3 were added into each well in the third and fourth groups (PGO and PGO-GS3 experimental groups), respectively. A total of 10 μL of PBS was added into each well in the first (blank group) and the second group (control group).

After incubation for 48 h, the cell viability was evaluated through CCK-8 (EnoGene Biotechnology Co., Ltd., Nanjing, Jiangsu Province, China) assay. Briefly, 10 μL CCK-8 solution and 90 μL cell culture medium were added into each well and incubated at 37 °C for 3 h. The light absorbance at the wavelength of 450 nm was measured using a microplate reader (Multiskan Go, Thermal fisher, Massachusetts, USA) for each well to calculate the cell viability. The cell survival rate was calculated using the equation below:

$$\mathrm{Sur}\;\%\;=\frac{\left(A_{s}-A_{b}\right)}{\left(A_{c}-A_{b}\right)}\times 100\%$$

where Sur % is the cell survival rate, A_s_ is the absorbance of the experimental group, A_b_ is the absorbance of the blank group, and A_c_ is the absorbance of the control group.

## Figures and Tables

**Table 1:** Density and porosity of hydrogels.

Hydrogel Samples	Density (g/cm^3^)	Porosity (%)
PGO	0.205 ± 0.009	40.59 ± 2.71
PGO-GS1	0.109 ± 0.003	60.77 ± 3.65
PGO-GS3	0.101 ± 0.019	62.28 ± 7.30
PGO-GS5	0.114 ± 0.012	58.58 ± 6.82

**Table 2:** The viability of cells incubated with hydrogel leach liquor for 48 h.

Group Name	Absorbance	Sur %
Blank group	0.35469	N/A
Control group	1.19736	100
PGO-GS3	0.99315	75.8
PGO	1.15768	95.3

## Data Availability

The data presented in this study are available on request from the corresponding author.

## Supplementary Materials

The following supporting information can be downloaded at: https://www.mdpi.com/article/10.3390/gels9020155/s1, Figure S1: The swelling ratios of hydrogels; Table S1: The conductivity of hydrogels at different degrees of bending.
