# Supplementary material for: Enhancing Conductivity and Self-Healing Properties of PVA/GEL/OSA Composite Hydrogels by GO/SWNTs for Electronic Skin
Source: Gels. 2023 Feb 15;9(2):155. doi: 10.3390/gels9020155 (PMC9956163; doi:10.3390/gels9020155)
Supplement: Supplementary file 1 [file gels-09-00155-s001.zip › gels-2222348-supplementary.pdf]

Enhancing Conductivity and Self-Healing Properties of PVA/GEL/OSA Composite Hydrogels by GO/SWNTs for Electronic Skin

Xiaohu Chen 1, 2 , Haonan Zhang 1,2, Jiashu Cui 1,2, Yanen Wang 1,2,*, Mingyang Li 1,2, Juan Zhang 1,2, Changgeng Wang 1,2, Zhisheng Liu 1,2 and Qinghua Wei 1,2,*

[1] Department of Indurstry and Engineering, School of Mechanical Engineering, Northwestern Polytechnical University, Xi'an 710072, China

[2] Bio-Additive Manufacturing University-Enterprise Joint Research Center of Shaanxi Province, Northwestern Polytechnical University, Xi'an, 710072, China

* Correspondence: wangyanen@nwpu.edu.cn (Y.W.); weiqinghua@nwpu.edu.cn (Q.W.); Tel./Fax: +86-029-88493232

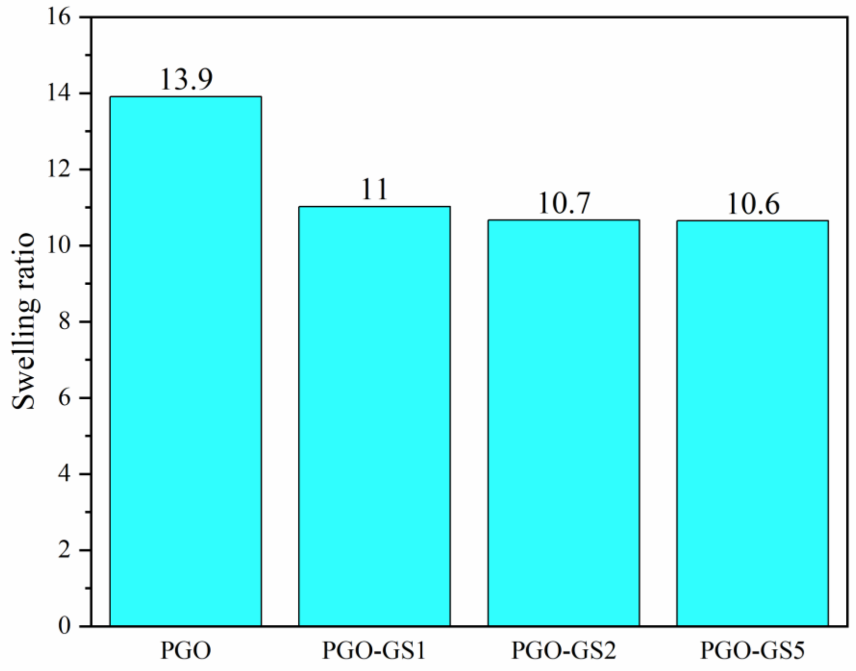

Figure S1. The swelling ratios of hydrogels.

Table S1 The conductivity of hydrogels at different degrees of bending.

Sample name	Parameter	The distance between electric clamps (5, 3, and 1 cm from left to right)		
PGO	Resistance (kΩ)	52.33	53.12	53.40
	Conductivity (mS/m)	19.11	18.82	18.73
PGO-GS3	Resistance (kΩ)	34.32	36.28	37.59
	Conductivity (mS/m)	29.13	27.56	26.60
